# Supplementary material for: Structural Analysis of Janus Tyrosine Kinase Variants in Hematological Malignancies: Implications for Drug Development and Opportunities for Novel Therapeutic Strategies
Source: Int J Mol Sci. 2023 Sep 26;24(19):14573. doi: 10.3390/ijms241914573 (PMC10572942; doi:10.3390/ijms241914573)
Supplement: Supplementary file 1 [file ijms-24-14573-s001.zip › ijms-2593664-supplementary.pdf]

Table S1. Human JAK Family functional domain regions

JAK	UniProtKD Entry	FERM Domain	SH2 Domain	Pseudokinase Domain	Kinase Domain
JAK1_HUMAN	P23458	34-420	439-544	583-855	875-1153
JAK2_HUMAN	O60674	37-380	401-482	545-809	849-1124
JAK3_HUMAN	P52333	24-356	375-475	521-781	822-1111
TYK2_HUMAN	P29597	26-431	437-534	589-867	892-1174

Table S2. Post-translation modification of JAK proteins

Post-translation modification	JAK1	JAK2	JAK3	TYK2
Phosphotyrosine	Y3, Y1034, Y1035, Y1125	Y119, Y373, Y570, Y813, Y868, Y966, Y972, Y1007, Y1008	Y105, Y190, Y238, Y399, Y633, Y637, Y738, Y762, Y785, Y824, Y904, Y929, Y939, Y980, Y981,	Y292, Y433, Y604, Y827, Y1054, Y1055
Phosphotyrosine by autocatalysis	-	Y119, Y868, Y966, Y972, Y1007, Y1008	Y105, Y190, Y238, Y399, Y633, Y637, Y738, Y762, Y785, Y824, Y904, Y929, Y939, Y980, Y981	Y1054
Phosphoserine	S228, S537, S857, S963	S523	S17, S20, S493, S783	S499, S525, S613, S884
Others	N-acetylmethionine in residue 1	-	-	Phospho-T879

Table S3: Full list of known JAK hematological malignancy variants. Acute lymphoblastic leukemia (ALL); Early T-cell precursor (ETP); Acute myeloid leukemia (AML); Polycythemia vera (PV); Myeloproliferative neoplasm (MPN); Essential thrombocythaemia (ET); Acute megakaryoblastic leukemia (AMKL); Adult T-cell leukemia/lymphoma (ATLL); T-cell pro-lymphocytic leukemia (T-PLL); Juvenile myelomonocytic leukemia (JMML); Natural killer/T-cell lymphoma (NKTCL); Idiopathic myelofibrosis (IMF); Down syndrome ALL (DS-ALL); Down syndrome therapy-related myelodysplatic syndrome (DS-TMD)

JAK1	Region	Original Amino acid	Position	Amino acid Change	Original Amino acid nature	New Amino acid nature	Hematopoietic Malignancy Subtype	Reference
	FERM	I	62	V	Nonpolar	Nonpolar	B-ALL; T-ALL	PMID: 18362173
		S	71	C	Polar	Polar	ETP-ALL	PMID: 23603912
		K	204	M	Positive	Nonpolar	B-ALL	PMID: 24154688
		R	360	W	Positive	Nonpolar	T-ALL	PMID: 18362173
		I	377	K	Nonpolar	Positive	ETP-ALL	PMID: 23603912
	Linker	V	427	M	Nonpolar	Nonpolar	Pediatric T-ALL	PMID: 26819051
	SH2	T	478	S	Polar	Polar	AML	PMID: 24843152
		S	512	L	Polar	Nonpolar	T-ALL	PMID: 18362173
	JH2	D	604	Y	Negative	Nonpolar	T-ALL	PMID: 21880637
		V	623	A	Nonpolar	Nonpolar	AML	PMID: 24843152
			L624-629W (L624_R629 >W)		indel*		ALL	PMID: 25504635,
		I	631	R	Nonpolar	Positive	ALL	PMID: 22237106
		I	631	G	Nonpolar	Nonpolar	ALL	PMID: 22237106
		A	634	D	Nonpolar	Negative	B-ALL; T-ALL	PMID: 18362173
		S	646	F	Polar	Nonpolar	ALL	PMID: 25504635
		S	646	P	Polar	Nonpolar	ALL	PMID: 21393331
		K	648	N	Positive	Polar	T-ALL	PMID: 21551237
		V	651	M	Nonpolar	Nonpolar	T-ALL	PMID: 24843152
		Y	652	H	Nonpolar	Positive	T-ALL	PMID: 24843152, PMID: 21393331
		L	653	F	Nonpolar	Nonpolar	Childhood ALL	PMID: 18362173
		V	658	F	Nonpolar	Nonpolar	ALL	PMID: 25504635, PMID: 21393331, PMID: 18559588, PMID: 16239216
		V	658	L	Nonpolar	Nonpolar	ALL	PMID: 21393331
		E	668	Q	Negative	Polar	Pediatric T-ALL	PMID: 26819051
		S	703	I	Polar	Nonpolar	ALL	PMID: 22237106, PMID: 21393331
		R	724	H	Positive	Positive	B-ALL; T-ALL	PMID: 18362173
		R	724	Q	Positive	Polar	B-ALL; T-ALL	PMID: 18362173
		R	724	S	Positive	Polar	B-ALL; T-ALL	PMID: 18362173
		F	734	L	Nonpolar	Nonpolar	T-ALL	PMID: 21393331
		L	783	F	Nonpolar	Nonpolar	T-ALL	PMID: 21393331, PMID: 18559588
	JH1	R	879	S	Positive	Polar	T-ALL	PMID: 18362173
		R	879	C	Positive	Polar	T-ALL	PMID: 18362173

		R	879	H	Positive	Positive	T-ALL	PMID: 18362173
		T	901	G	Polar	Nonpolar	T-ALL	PMID: 26819051
		K	908	T	Positive	Polar	Pediatric T-ALL	PMID: 26819051

JAK2	Region	Original Amino acid	Position	Amino acid Change	Original Amino acid nature	New Amino acid nature	Hematopoietic Malignancy Subtype	Reference
		E	61	K	Negative	Positive	Putative primary erythrocytosis	PMID: 27651169
		T	108	A	Polar	Nonpolar	Found as germline mutation in V617F-positive PV patient	PMID: 27647865
	FERM	E	177	V	Negative	Nonpolar	Putative primary erythrocytosis	PMID: 27651169
		G	276	A	Nonpolar	Nonpolar	Putative primary erythrocytosis	PMID: 27651169
		R	340	Q	Positive	Polar	PV	PMID: 20417861
	Linker	L	393	V	Nonpolar	Nonpolar	Found as germline mutation in V617F-positive PV patient	PMID: 27647865
		T	514	M	Polar	Nonpolar	MPNs	PMID: 19074595
		N	533	I	Polar	Nonpolar	PV (together with K539L)	PMID: 21864276
		N	533	Y	Polar	Nonpolar	PV (together with K539L)	PMID: 21864276
		M	535	I	Nonpolar	Nonpolar	AMKL	PMID: 19638629
	Linker	K	539	L	Positive	Nonpolar	MPNs	PMID: 17267906
		I	540	T	Nonpolar	Polar	PV	PMID: 24843152
			538-547				MPNs	PMID: 24843152
		D	544	G	Negative	Nonpolar	PV	PMID: 24843152
		L	545	S	Nonpolar	Polar	PV	PMID: 24843152
		F	547	L	Nonpolar	Nonpolar	PV	PMID: 24843152
		F	556	L	Nonpolar	Nonpolar	MPNs	PMID: 21864276
		R	564	Q	Positive	Polar	Hereditary ET	PMID: 24381227
		V	567	A	Nonpolar	Nonpolar	MPNs	PMID: 21864276
		H	587	N	Positive	Polar	MPNs	PMID: 21864276
		S	591	L	Polar	Nonpolar	MPNs	PMID: 21864276
		H	606	Q	Positive	Polar	MPNs	PMID: 21864276
		K	607	N	Positive	Polar	AML	PMID: 21864276
		H	608	Y	Positive	Nonpolar	MPNs	PMID: 21864276
	JH2	L	611	S	Nonpolar	Polar	ALL	PMID: 21864276
		V	617	F	Nonpolar	Nonpolar	MPNs	PMID: 15781101, PMID: 15793561, PMID: 15858187, PMID: 15837627
		V	617	I	Nonpolar	Nonpolar	Hereditary thrombocythemia	PMID: 22397670, PMID: 23535062
		C	618	R	Polar	Positive	MPNs	PMID: 22960131
		D	620	E	Negative	Negative	MPNs	PMID: 16871281
		L	624	P	Nonpolar	Nonpolar	MPNs	PMID: 21864276
		I	645	V	Nonpolar	Nonpolar	MPNs	PMID: 21864276
		I	682	F	Nonpolar	Nonpolar	ALL	PMID: 19470474
		R	683	S	Positive	Polar	MPNs	PMID: 19470474, PMID: 18805579
		R	683	G	Positive	Nonpolar	MPNs	PMID: 19470474, PMID: 18805579

		S	755	R	Polar	Positive	Hereditary thrombocythemia (together with R938Q in cis)	PMID: 24398328
		Y	813	D	Nonpolar	Negative	IMF	PMID: 24843152
	Linker	E	846	D	Negative	Negative	Germ-line mutation found in erythrocytosis and megakaryocytic atypia	PMID: 27389715
		R	867	Q	Positive	Polar	ALL, hereditary thrombocythemia	PMID: 25504635
		D	873	N	Negative	Polar	ALL	PMID: 19470474
		T	875	N	Polar	Polar	AMKL	PMID: 24843152
	JH1	P	933	R	Nonpolar	Positive	ALL	PMID: 19470474
		R	938	Q	Positive	Polar	Hereditary thrombocythemia (together with S755R in cis)	PMID: 24398328
		R	1063	H	Positive	Positive	Germ-line mutation found in erythrocytosis and megakaryocytic atypia	PMID: 27389715
		N	1108	S	Polar	Polar	PV	PMID: 24843152

**JAK3**	**Region**	**Original Amino acid**	**Position**	**Amino acid Change**	**Original Amino acid nature**	**New Amino acid nature**	**Leukemia Subtype**	**Reference**
		G	62	S	Nonpolar	Polar	AML	PMID: 20400977
		I	87	T	Nonpolar	Polar	DS-TMD/AML	PMID: 14615376, PMID: 18397343
		P	132	T	Nonpolar	Polar	AMKL	PMID: 16843266, PMID: 21599579
		P	132	A	Nonpolar	Nonpolar	AMKL	PMID: 16843266, PMID: 21599579
	FERM	P	151	R	Nonpolar	Positive	DS-TMD, AMLK	PMID: 11668610, PMID: 17456055
		L	156	P	Nonpolar	Nonpolar	ATLL	PMID: 21821710
		R	172	Q	Positive	Polar	ATLL	PMID: 21821710
		E	183	G	Negative	Nonpolar	ATLL	PMID: 21821710
		R	272	H	Positive	Positive	T-ALL	PMID: 25193870
		Q	283	H	Polar	Positive	T-ALL	PMID: 26206799
	SH2	R	403	H	Positive	Positive	T-ALL	PMID: 25193870, PMID: 9354668
		Q	501	H	Polar	Positive	AMKL	PMID: 24843152, PMID: 18397343
	Linker	Q	507	P	Polar	Nonpolar	T-PLL	PMID: 24446122
		M	511	I	Nonpolar	Nonpolar	T-PLL, AML, JMML, NKTCL	PMID: 25193870, PMID: 24446122
		A	572	V	Nonpolar	Nonpolar	AMKL	PMID: 24843152, PMID: 25193870,
		A	572	T	Nonpolar	Polar	AMKL	PMID: 24843152, PMID: 25193870,
		A	573	V	Nonpolar	Nonpolar	DS-ALL, DS AMKL, NTCL	PMID: 24843152, PMID: 25193870, PMID: 19139084, PMID: 29046866, PMID: 28284718
		M	576	L	Nonpolar	Nonpolar	Adult non-DS AMKL	PMID: 17252020
		H	583	Y	Positive	Nonpolar	NTCL	PMID: 14615376, PMID: 28284718
	JH2	G	589	D	Nonpolar	Negative	NTCL	PMID: 28284718
			592		Deletion*		AMKL/TMD	PMID: 17456055
		A	593	R	Nonpolar	Positive	AMKL/TMD	PMID: 17456055
		A	593	T	Nonpolar	Polar	AMKL/TMD	PMID: 17456055
		R	657	Q	Positive	Polar	T-PLL, AML, JMML, NKTCL	PMID: 24843152, PMID: 24446122
		R	657	H	Positive	Positive	T-PLL, AML, JMML, NKTCL	PMID: 24843152, PMID: 24446122
		R	657	W	Positive	Nonpolar	T-PLL, AML, JMML, NKTCL	PMID: 24843152, PMID: 24446122
		V	674	A	Nonpolar	Nonpolar	T-ALL	PMID: 24446122, PMID: 22425895, PMID: 26446793

		V	674	F	Nonpolar	Nonpolar	T-ALL	PMID: 24446122, PMID: 22425895, PMID: 26446793
		V	678	L	Nonpolar	Nonpolar	T-ALL	PMID: 25193870, PMID: 24446122
		V	678	M	Nonpolar	Nonpolar	T-ALL	PMID: 25193870, PMID: 24446122
		V	722	I	Nonpolar	Nonpolar	AMKL, NKTCL	PMID: 16843266, PMID: 10982185
		V	765	D	Nonpolar	Negative	JMML	PMID: 23832011
	Linker	S	789	P	Polar	Nonpolar	Childhood ALL	PMID: 19470474, PMID: 29046866
		Y	824	D	Nonpolar	Negative	T-PLL	PMID: 24048415
		L	857	Q	Nonpolar	Polar	T-ALL/T-PLL/JMML	PMID: 25193870, PMID: 26446793
		L	857	P	Nonpolar	Nonpolar	T-ALL/T-PLL/JMML	PMID: 25193870, PMID: 26446793
		Q	865	E	Polar	Negative	T-ALL	PMID: 26206799
		P	906	S	Nonpolar	Polar	ALL	PMID: 26446793
	JH1	R	918	C	Positive	Polar	AML	PMID: 20400977, PMID: 29046866
		R	925	S	Positive	Polar	T-ALL	PMID: 25193870
		E	958	K	Negative	Positive	T-ALL, JMML	PMID: 26206799, PMID: 26446793, PMID: 23832011, PMID: 26980750
		Q	988	P	Polar	Nonpolar	T-ALL	PMID: 24367274
		L	1017	M	Nonpolar	Nonpolar	CML	PMID: 20400977
		E	1106	G	Negative	Nonpolar	T-ALL	PMID: 25193870

TYK2	Region	Original Amino acid	Position	Amino acid Change	Original Amino acid nature	New Amino acid nature	Leukemia Subtype	Reference
		G	36	D	Nonpolar	Negative	T-ALL (MOLT-16 cell line)	PMID: 23471820
		S	47	N	Polar	Polar	T-ALL (MOLT-16 cell line)	PMID: 23471820
	FERM	G	363	S	Nonpolar	Polar	AML	PMID: 18270328
		R	425	H	Positive	Positive	T-ALL (MOLT-16 cell line)	PMID: 23471820
		I	684	S	Nonpolar	Polar	T-ALL	PMID: 23471820
		R	703	W	Positive	Nonpolar	AML	PMID: 18270328
	JH2	V	731	I	Nonpolar	Nonpolar	ALL (RPMI-8402 cell line)	PMID: 23471820
		P	760	L	Nonpolar	Nonpolar	ALL (germline)	PMID: 27733777
		G	761	V	Nonpolar	Nonpolar	ALL (germline)	PMID: 27733777
		M	926	V	Nonpolar	Nonpolar	Germline mutation in ALL, but probably not deleterious	PMID: 27733777
		E	957	D	Negative	Negative	ALL (MOLT-4 cell line)	PMID: 23471820
	JH1	A	1016	S	Nonpolar	Polar	AML	PMID: 18270328
		R	1027	H	Positive	Positive	ALL (MOLT-16 and CCRF-CEM cell lines)	PMID: 23471820

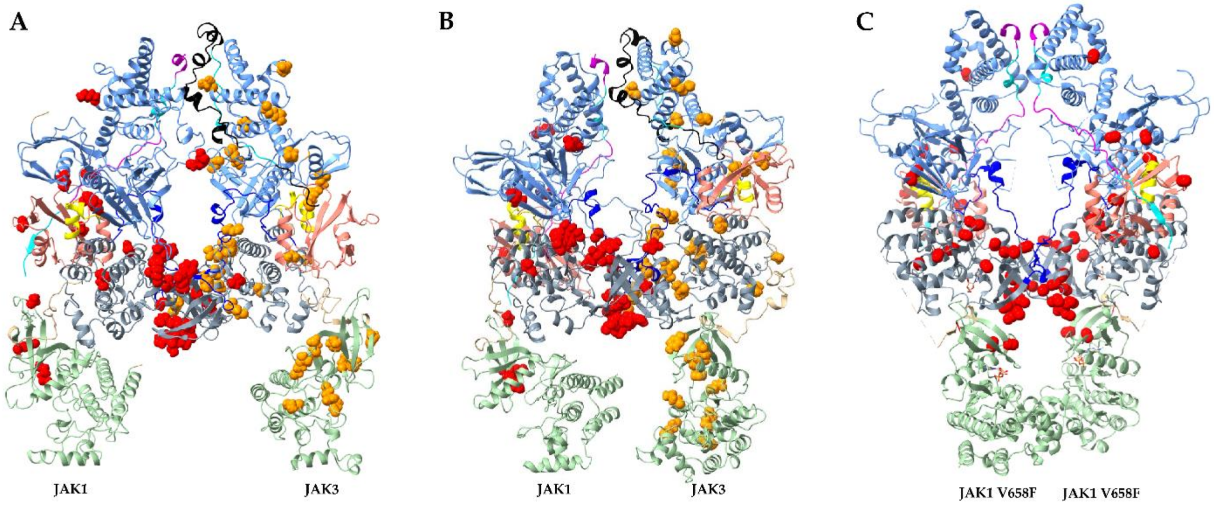

Figure S1. *Comparison of SWISS-MODEL and Alphafold2 JAK1-JAK3 dimer structures*. JAK FERM domain (light blue), FERM-SH2 linker (yellow), SH2 domain (pink), SH2-JH2 linker (royal blue), JH2 domain (gray), JH2-JH1 linker (beige), JH1 domain (green). JAK1 and JAK3 residues, red and orange spheres respectively, related to hematologic malignancies are shown. A) SWISS-MODEL structure obtained using PDB 7T6F for both JAK1 and JAK3. B) Alphafold2 Multimer V3 model obtained using PDB 7T6F as a template. C) Published structure of JAK1 V658F (PDB 8EWY) homodimer in transactivation process.
